# Dietary fruit, vegetable, fat, and red and processed meat intakes and Barrett’s esophagus risk: a systematic review and meta-analysis

**DOI:** 10.1038/srep27334

**Published:** 2016-06-03

**Authors:** Zhanwei Zhao, Zhongshu Pu, Zifang Yin, Pengfei Yu, Yiming Hao, Qian Wang, Min Guo, Qingchuan Zhao

**Affiliations:** 1 Xijing Hospital of Digestive Diseases, The Fourth Military Medical University, 127 Changle Western Road, Xi’an, Shaanxi Province 710032, China; 2 Department of Epidemiology, School of Public Health, The Fourth Military Medical University, 127 Changle Western Road, Xi’an, Shaanxi Province 710032, China.; 3 Shaanxi Maternal and Child Health Hospital, Shaanxi Province, China

## Abstract

The relationships between dietary fruit, vegetable, fat, and red and processed meat intakes and Barrett’s esophagus (BE) risk remain inconclusive. We conducted a systematic review and meta-analysis to summarize the available evidence on these issues. PubMed, EMBASE and the Cochrane Library were searched for studies published from inception through October 2015. A total of eight studies were included in this analysis. Fruit intake was not associated with BE risk (OR = 0.65, 95% CI = 0.37–1.13), but vegetable intake was strongly associated with BE risk (OR = 0.45, 95% CI = 0.29–0.71). Saturated fat, red meat and processed meat intakes were not associated with BE risk with OR = 1.25 (95% CI = 0.82–1.91), OR = 0.85 (95% CI = 0.61–1.17) and OR = 1.03 (95% CI = 0.73–1.46), respectively. Dietary vegetable not fruits intake may be associated with decreased BE risk. Fat and red and processed meat intakes may not contribute to an increased BE risk. Well-designed, large prospective studies with better established dose-response relationships are needed to further validate these issues.

Barrett’s esophagus (BE) is considered to be the strongest risk factor^1^ and the only known precursor for esophageal adenocarcinoma (EAC), with a thirty- to forty-fold increased risk^2^,^3^. EAC is one of the most lethal malignancies in the Western world, with a rapidly rising incidence^1^,^4^. In the US, EAC has increased at least six-fold over the last four decades^5^,^6^,^7^.

BE is defined as the presence of columnar-type mucosa of the esophagus on endoscopy and pathology, and BE is also recognized as a specialized intestinal metaplasia by histology of the esophagus in most studies^1^,^5^,^8^. Many studies have shown the wide and increasing prevalence of BE throughout the world^9^,^10^,^11^. Although the risk factors of BE have been reported, including gastro-esophageal reflux disease (GERD), obesity, *H. pylori* infection, smoking status and alcohol intake, the causes and pathogenesis of the high prevalence of BE are still unclear^9^,^12^,^13^,^14^,^15^,^16^.

There are some studies^17^,^18^ reporting the role of other factors, such as dietary fruits, vegetables, fat, and red and processed meat intakes, for BE risk; however, the associations between these factors and BE risk remain uncertain because of the sparse studies and limited epidemiologic data. In particular, there has been no systematic review or meta-analysis on these issues as of yet. Therefore, we conducted the first systematic review and meta-analysis of the available evidence to address these important questions on the relationships of dietary fruit, vegetable, fat, and red and processed meat intakes with BE risk.

## Methods

### Selection criteria

1. Studies unrelated to our topics were excluded.
2. Case-control studies and cohort studies were included.
3. The patients with BE were all diagnosed via endoscopy and biopsy. The histological feature, which was not consistent with the diagnostic gold standard, was excluded.
4. Data that were incomplete or could not be combined were excluded.
5. Narrative reviews, systematic reviews and meta-analyses were excluded.
6. Cases in which only comments, case reports, editorials, letters and the abstract could be obtained were excluded.
7. The most recent studies, the most samples, and the quality studies that included reports with the same patients were selected.
8. Red and processed meats in this study included beef, corned beef, beefburgers, veal, bacon, bacon rashers, luncheon meat, lamb, mutton, ham, sausage, salami, hot dogs, souse meat, smoked meat, salted meat and barbecued meat.
9. Poultry, chicken, fish and other white meats were excluded.

### Search strategy

We searched PubMed, EMBASE and the Cochrane Library for studies of fruits, vegetables, fat, and red and processed meats and BE published from inception through October 2015. The following search terms were used: *“fruit”, “fruits”, “vegetable”, “vegetables”, “fat”, “meat”, “red meat”, “processed meat”, “beef”, “corned beef”, “beefburgers”, “veal”, “bacon”, “bacon rashers”, “pork”, “lamb”, “mutton”, “luncheon meat”, “ham”, “sausage”, “salami”, “hot dogs”, “sauce meat”, “smoked meat” “salted meat”, “barbecued meat”* in combination with *“Barrett’s esophagus/oesophagus”, “gastrointestinal tract”, “metaplasia”, “precancerous/neoplasia/canceration/cancerization”, “carcinogenesis/tumorigenesis”, “esophageal cancer/carcinoma/adenomas/adenocarcinoma”*. Reference lists in the included studies were also searched manually to identify additional literature. The two sets of keywords were combined individually, and the eligibility criterion was independently judged by two authors (Zhanwei Zhao and Zhongshu Pu). In case of disagreement between the two authors, the third author (Qingchuan Zhao) made a consensus decision. The language of all studies was limited to English only. The studies were also limited to only those in humans.

### Definitions and Standardizations

#### Barrett’s esophagus (BE)

BE is defined as the presence of columnar-type mucosa in the esophagus on endoscopy and pathology. Subsequently, BE is also recognized as a specialized intestinal metaplasia by histology of the esophagus in most studies^1^,^5^,^8^. Barrett’s columnar epithelium is considered to be a marker for severe reflux and a precursor to adenocarcinoma of the esophagus^5^,^19^.

#### Red and processed meats

In this study, red and processed meats included beef, corned beef, pork, lamb, mutton, beefburgers, veal, bacon, bacon rashers, luncheon meat, ham, sausage, salami, hot dogs, souse meat, smoked meat, salted meat and barbecued meat.

#### Study quality

Study quality in this meta-analysis was assessed using the Newcastle-Ottawa Scale (NOS)^20^. The range of NOS was 0–10 stars, which was judged in three parts, including the elucidation of exposure or the outcomes of interest for case-control or cohort studies, the selection of the study populations and the comparability of the populations. Two authors (Zhanwei Zhao and Zhongshu Pu) independently assessed the quality of the studies, and a consensus decision was made regarding any discrepancies in interpretations by the third author (Qingchuan Zhao).

#### Data Extraction

A data extraction sheet was set up to enter data from each study, including the first author, year of publication, country, study type, case/control, study population, method of dietary assessment, type of dietary exposure measured, meat exposure categories, NOS score and controlled variables (Table 1). The controlled variables were specifically listed in Table 2.

### Statistical analysis

The data were collected and extracted using SPSS 17.0 (Chicago, Illinois, USA). RevMan 5.3 (The Cochrane Collaboration, Oxford, UK) and STATA, version 12.1 (STATA Corporation, College Station, TX) software were used for data synthesis and analysis. Heterogeneity was detected using the *I*^2^ statistic (25%, 50% and 75% meant low, moderate and high heterogeneity, respectively; in this analysis, *I*^2^ < 50% was considered as having low heterogeneity among studies, while *I*^2^ > 50% was considered as having high heterogeneity)^21^. A fixed-effects model was used if there was no heterogeneity among the studies, and a random-effects model was used if there was heterogeneity among the studies. Publication bias was tested using Begg’s test.

## Results

### Literature selection, study characteristics and quality scores

**Figure 1:**
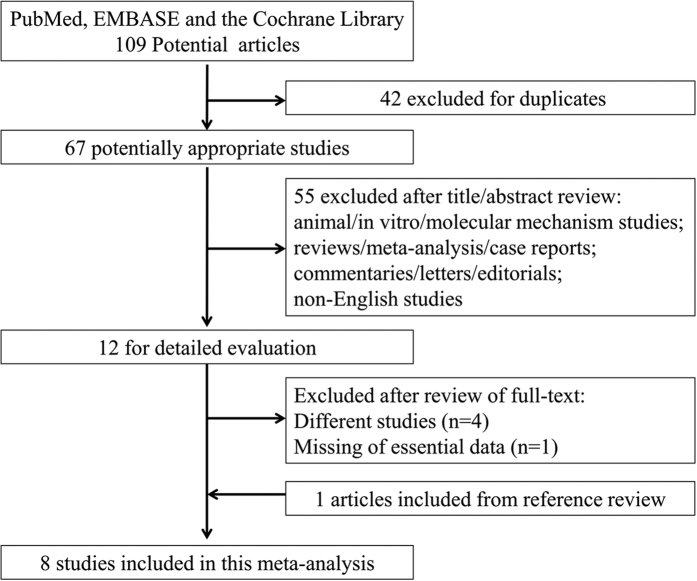
Flowchart of the process for the identification of relevant studies.

Figure 1 shows the flowchart of the search strategy for selecting eligible studies. A total of 109 studies were initially identified for this meta-analysis. Forty-two studies were excluded for duplication, and 67 studies were selected for further consideration. Among them, 55 studies were excluded after reviewing the titles and abstracts. Finally, 8 studies met the eligibility criteria after excluding the different studies (n = 4) and missing data (n = 1), and including one study from reference review.

The 8 selected studies included 6 case-control studies and 2 cohort studies with 858 cases of fruit intake, 858 cases of vegetable intake, 667 cases of fat intake, 412 cases of red meat intake and 633 cases of processed meat intake (Table 1). The NOS scores of quality of the included studies are listed in Table 1. The controlled variables are specifically listed in Table 2.

### Fruit intake and BE risk

**Figure 2:**
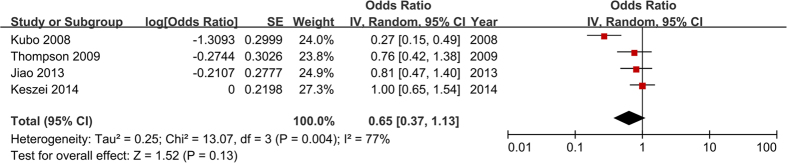
Estimates (95% CI) of fruit intake (highest versus lowest category) and BE risk. There was no association between fruit intake and BE risk (*P* = 0.13).

The ORs have been adjusted for the potential multivariable confounders, including age, sex, ethnicity, energy intake, body mass index, waist-to-hip ratio, smoking, alcohol, education, medication use, gastro-esophageal reflux and vitamins. Figure 2 showed that the pooled OR was 0.65 (95% CI = 0.37–1.13) with significant heterogeneity (*P* = 0.004, *I*^ 2 ^ = 77%). No statistical evidence of publication bias was observed from Begg’s test (*P* = 0.089). These results suggest that fruit intake may be not significantly associated with BE risk.

### Vegetable intake and BE risk

**Figure 3:**
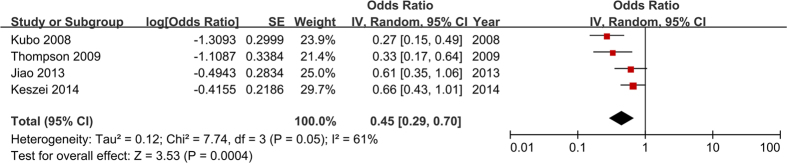
Estimates (95% CI) of vegetable intake (highest versus lowest category) and BE risk. There was an association between vegetable intake and BE risk (*P* = 0.0004).

The ORs (95% CI) of each study and the pooled OR (95% CI) for the highest versus lowest category are listed in Fig. 3. The ORs were adjusted for the confounders listed above. The pooled OR was 0.45 (95% CI = 0.29–0.71) with heterogeneity (*P* = 0.05, *I*^ 2^ = 61%) with no publication bias according to Begg’s test (*P* = 0.308). The results indicate that vegetable intake may have a strong inverse association with BE risk (with a 55% decreased risk of BE).

### Fat intake and BE risk

**Figure 4:**
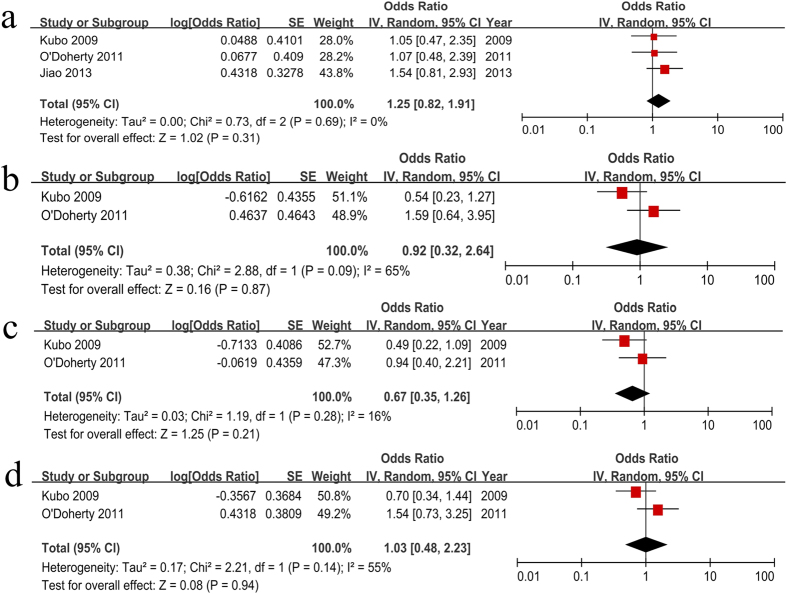
Subtypes of fat intake (highest versus lowest category) and BE risk. (**a**) saturated fat (*P* = 0.31); (**b**) monounsaturated fat (*P* = 0.87); (**c**) polyunsaturated fat (*P* = 0.21); (**d**) cholesterol (*P* = 0.94). There were no associations between fats intake and BE risk.

The ORs were adjusted for the confounders listed above and Helicobacter pylori infection. The pooled OR of saturated fat intake was 1.25 (95% CI = 0.82–1.91, Fig. 4a) with no significant heterogeneity (*P* = 0.69, *I*^ 2^ = 0%) and no publication bias according to Begg’s test (*P* = 0.540). There were two case-control studies for monounsaturated fat (Fig. 4b) and the pooled OR was 0.92 (95% CI = 0.32–2.64) with heterogeneity (*P* = 0.09, *I*^ 2^ = 65%). Begg’s test showed no statistical evidence of publication bias (*P* = 1.000). The pooled OR of polyunsaturated fat intake was 0.67 (95% CI = 0.35–1.26) with no heterogeneity (*P* = 0.28, *I*^ 2^ = 16%, Fig. 4c) and no publication bias (Begg’s test, *P* = 1.000). Additionally, OR = 1.03 (95% CI = 0.48–2.23) indicated that there was no association between cholesterol intake and BE risk (Fig. 4d). There was no analysis for other types of fat, such as trans fat and omega 3 because of limited data. These results suggest that fat intake may be not associated with BE risk.

### Red and processed meat intake and BE risk

**Figure 5:**
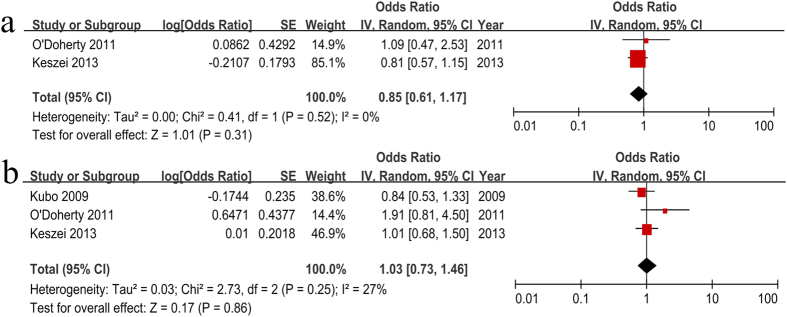
Estimates (95% CI) of red and processed meat intake (highest versus lowest category) and BE risk. (**a**) red meat; (**b**) processed meat. There were no associations between red (*P* = 0.31) and processed (*P* = 0.86) meat intake and BE risk.

The ORs (95% CI) of each study and the pooled OR (95% CI) for the highest versus lowest category are listed in Fig. 5a,b. The ORs were adjusted for potential multivariable confounders, including age, sex, energy intake, body mass index, waist-to-hip ratio, physical activity, smoking, alcohol, education, medication use, gastro-esophageal reflux and *H. pylori*. A pooled analysis yielded the findings that red meat consumption was no associated with BE risk (OR = 0.85, 95% CI = 0.61–1.17) with no significant heterogeneity (*P* = 0.52, *I*^ 2^ = 0%, Fig. 5a). Additionally, there was no association between processed meat consumption and BE risk (OR = 1.03, 95% CI = 0.73–1.46) with no heterogeneity (*P* = 0.25, *I*^ 2^ = 27%). These results suggest that red and processed meat consumption may be not associated with BE risk.

### Dose-response meta-analysis

We also performed a dose-response meta-analysis to evaluate the dose-response associations between dietary fruit and vegetable^17^,^19^,^22^, saturated fat^23^,^24^, monounsaturated fat^23^,^25^ and polyunsaturated fat^23^,^25^ intakes and BE risk. It was not possible to analyze dose-response relationships for cholesterol, red meat and processed meat due to limited data.

The dose-response analysis showed that BE risk decreased 16% (OR: 0.84, 95% CI 0.78–0.91) per unit increase (serving/day) with vegetable intake. There were no significant changes of BE risk per unit increase (serving/day) with fruit intake (OR = 0.66, 95% CI = 0.39–1.14) and per unit increase (gram/day) with saturated fat intake (OR: 1.03, 95% CI 0.99–1.07), monounsaturated fat intake (OR: 1.15, 95% CI 0.63–2.09) or polyunsaturated fat intake (OR: 0.70, 95% CI 0.40–1.25).

## Discussion

Studies have explored and reported the possible relationships and mechanisms supporting the idea that high fruit and vegetable intakes reduce BE risk. Dietary fruits and vegetables may be inversely associated with BE risk, which may influence the early carcinogenesis of EAC^19^. Fruit intake is inversely associated with BE risk and may influence the process of carcinogenesis of EAC^19^. Keszei *et al*.^26^ conducted a large Dutch cohort study and found that vegetable consumption may prevent the risk of BE, and intriguingly, this effect was different in men and women. However, findings from another study supported the idea that increased intake of fruits and vegetables is associated with a lower BE risk in both men and women^27^.

Polyunsaturated fat and omega 3 intakes show inverse associations with BE risk that are stronger for long-segment BE^23^; however, higher total fat and saturated fat intakes have been reported to be associated with significantly increased BE risk (3^rd^ compared with 1^st^)^24^. In contrast, some studies have reported inconsistent findings in terms of associations between total fat^23^,^25^ and BE risk. Thus, these issues need to be examined by further investigations.

Recently, meats, particularly red and processed meat intakes, have been reported as risk factors for oral cavity and oropharynx cancer^28^,^29^, EAC^30^,^31^,^32^, gastric cancer^33^,^34^, colorectal cancer^35^,^36^,^37^, pancreatic cancer^38^, hepatocellular carcinoma^39^, breast cancer^40^,^41^, lung cancer^42^,^43^, renal cancer^44^, bladder cancer^45^,^46^, ovarian cancer^47^, brain tumors^48^, glioma^49^, non-Hodgkin lymphoma^50^, type 2 diabetes^51^, stroke^52^ and coronary heart disease^53^. Red and processed meats are one of the major sources of nitrate and N-nitroso compounds, which are considered to be carcinogenic in humans and risk factors of BE^54^; however, it is worth mentioning that not all cooked meats increase BE risk. Existing studies have suggested that there was no obvious association between well-cooked meats and BE risk^55^,^56^. Moreover, the results of some studies have shown that red and processed meat intakes were not associated with BE risk^18^,^25^.

Although some studies have reported that dietary fruit, vegetable, fat, and red and processed meat intakes were associated with BE risk, the related evidence is sparse and inconsistent, and there had been no published meta-analysis. This study focuses on these issues for the first time and provides reliable evidence to date.

Eight hundred fifty-eight cases of fruit intake, 858 cases of vegetable intake, 667 cases of fat intake, 412 cases of red meat intake and 633 cases of processed meat intake were included in this analysis. Our findings indicated that dietary vegetable rather than fruit intake was associated with significantly reduced BE risk (with 55% lower risk). Furthermore, the dose-response analysis suggested that the increases in vegetable [per unit increase (serving/day)] intake was significantly associated with a 16% decreased risk of BE.

Figures 4a–d indicated that fats, including saturated fat, monounsaturated fat, polyunsaturated fat and cholesterol intakes, were not associated with BE risk. The dose-response analysis also yielded similar results. Other types of fat intake were not analyzed due to the limited data.

Although previous studies have reported that red and processed meat intakes were associated with a significantly higher incidence of BE^54^, our analysis suggested that there are inconsistent results (Fig. 5a,b). In addition, the dose-response analysis also did not indicate significant associations between red and processed meat intakes and BE risk.

Overall, these results suggest that essential moderate vegetable intake was associated with decreased BE risk, which may be important for the prevention of EAC. Dietary fruit, fat and red and processed meat intakes may be not associated with BE risk. Nonetheless, these results should be treated with caution, and more high-quality designs are needed to further validate these findings because of the limited studies included, the heterogeneity among the studies and the influence of potential confounders (listed in the following section on limitations).

### Study strengths and limitations

Because there is no previously published meta-analysis that has evaluated the overall effects of fruit, vegetable, fat, and red and processed meat intakes on BE risk, a quantitative synthesis of the eligible data from included studies was required to provide important evidence on the associations. The dose-response analysis was also conducted to further assess these associations.

However, the limitations of the present meta-analysis must be taken into consideration. First, various potential factors may contribute to the heterogeneity of the included observational studies. However, the ORs of all these studies were adjusted for potential multivariable confounders, including age, sex, ethnicity, energy intake, body mass index, waist-to-hip ratio, physical activity, smoking, alcohol, education, medication use, gastro-esophageal reflux *H. pylori* and vitamins. Second, the quality of some studies was not high, despite meeting the criteria, and the sample size was not large. Third, there were five studies performed in the USA, two studies performed in the Netherlands and one study performed in the UK (Table 2), which may be related to the heterogeneity of statistical generalizability to some degree. Thus, more multicenter studies should be performed in other countries and regions. Fourth, because the results in this meta-analysis were only based on the diagnosis of BE, the limited data available should not be used to infer conclusions about other gastrointestinal lesions, particularly EAC. Fifth, because of the small number of studies, our analysis did not perform any subgroup analyses on the types of common fruits, such as apple, pear, orange and banana, and vegetables, such as dark green vegetables, leafy vegetables, starchy vegetables, allium, garlic, pepper and legumes^17^,^26^. Sixth, our study did not investigate the associations between BE risk and other dietary factors, such as fish, poultry meat^18^, other white meat^25^, cooking techniques, nitrates from pesticides on fruits and vegetables, heme iron from meat, dairy products, sugar, protein, dietary antioxidants and mineral intake. Further studies with multifactorial subgroup analyses are needed to provide more complete data. Lastly, the study type, publication year, geographic location, sample size, type of dietary exposure, dietary exposure category and quality of the studies may lead to bias.

## Conclusions

This is the first systematic review and meta-analysis that examined the associations between fruit, vegetable, fat, and red and processed meat intakes and BE risk. Dietary vegetable intake may be significantly associated with a decreased risk of BE. Dietary fruit, fat and red and processed meat intakes do not contribute to an increased BE risk. Well-designed, large, prospective studies with better established dose-response relationships are needed to further validate these findings.

## Additional Information

**How to cite this article**: Zhao, Z. *et al*. Dietary fruit, vegetable, fat, and red and processed meat intakes and Barrett’s esophagus risk: a systematic review and meta-analysis. *Sci. Rep.* **6**, 27334; doi: 10.1038/srep27334 (2016).

## Figures and Tables

**Table 1:** Baseline characteristics of the included studies.

First author, year, country	Study type	Case/ control	Study population	Method of dietary assessment	Type of dietary exposure	Dietary exposure categories	NOS score
Fruits							
Kubo USA^19^	case-control	296/308	M&W	FFQ	Total	Quartile	8
Thompson USA^22^	case-control	170/182	M&W	FFQ	Total	Tertile	9
Jiao USA^17^	case-control	151/777	M&W	FFQ	Total	Tertile	8
Keszei N^26^	cohort	241	M&W	FFQ	Total	Quintile	7
Vegetables							
Kubo USA^19^	case-control	296/308	M&W	FFQ	Total	Quartile	8
Thompson USA^22^	case-control	170/182	M&W	FFQ	Total	Tertile	9
Jiao USA^17^	case-control	151/777	M&W	FFQ	Total	Tertile	8
Keszei N^26^	cohort	241	M&W	FFQ	Total	Quintile	7
Fat							
Kubo USA^23^	case-control	296/309	M&W	FFQ	Total	Quartile	8
O’Doherty UK^25^	case-control	220/256	M&W	FFQ	Total	Quartile	8
Jiao USA^24^	case-control	151/777	M&W	FFQ	Saturated	Tertile	8
Red meats							
O’Doherty UK^25^	case-control	214/256	M&W	FFQ	Total and fresh	Quartile	8
Keszei 2013 N^18^	cohort	198	M&W	FFQ	Total	Tertile	7
Processed meats							
Kubo USA^23^	case-control	221/219	M&W	FFQ	Barbecued	Quartile	8
O’Doherty UK^25^	case-control	214/256	M&W	FFQ	Total	Quartile	8
Keszei N^18^	cohort	198	M&W	FFQ	Total	Tertile	7

N: Netherlands; FFQ: food frequency questionnaire; M: men; W: women.

**Table 2:** Controlled variables of the included studies.

First Author	Age	Sex	Ethnicity	Energy intake	BMI	WHR	Physical activity	Medication use	Alcohol intake	Smoking status	Education level	GER	*H. pylori*	V
Kubo USA^19^	√	√	√	√	√	√		√	√	√	√	√		√
Kubo USA^23^	√	√	√	√	√	√		√	√	√	√	√	√	√
Thompson USA^22^	√	√	√	√	√	√				√	√	√		√
O’Doherty UK^25^	√	√		√	√		√	√	√	√	√	√	√	
Jiao USA^24^	√	√	√	√	√	√	√	√	√	√	√	√		
Jiao USA^17^	√	√	√	√	√	√	√	√	√	√	√	√		√
Keszei N^18^	√	√		√	√		√	√	√	√	√			
Keszei N^26^	√	√		√	√		√	√	√	√	√			√

N: Netherlands; BMI: body mass index (kg/m^2^); WHR: waist-to-hip ratio; GER: gastro-esophageal reflux; V: vitamin.
